# Nucleic Acid Drugs—Current Status, Issues, and Expectations for Exosomes

**DOI:** 10.3390/cancers13195002

**Published:** 2021-10-05

**Authors:** Yoji Yamada

**Affiliations:** Research Management Office, Research Unit, R&D Division, Kyowa Kirin Co. Ltd., 1-9-2, Otemachi, Chiyoda-ku, Tokyo 100-0004, Japan; yoji.yamada.yh@kyowakirin.com

**Keywords:** nucleic acid drugs, modality, clinical application, rare disease, antisense oligonucleotide, siRNA, miRNA, exosome

## Abstract

**Simple Summary:**

Nucleic acid drugs provide novel therapeutic modalities with characteristics that differ from those of small molecules and antibodies. In this review, I focus on the various mechanisms through which nucleic acid drugs act on, the status of their clinical development, and discuss several hurdles that need to be surmounted. In addition, by listing examples of how the progress in exosome biology can lead to the solution of problems in nucleic acid drug therapy, I hope that many more nucleic acid drugs including anticancer drugs will be developed in the future.

**Abstract:**

Nucleic acid drugs are being developed as novel therapeutic modalities. They have great potential to treat human diseases such as cancers, viral infections, and genetic disorders due to unique characteristics that make it possible to approach undruggable targets using classical small molecule or protein/antibody-based biologics. In this review, I describe the advantages, classification, and clinical status of nucleic acid therapeutics. To date, more than 10 products have been launched, and many products have been tested in clinics. To promote the use of nucleic acid therapeutics such as antibodies, several hurdles need to be surmounted. The most important issue is the delivery of nucleic acids and several other challenges have been reported. Recent advanced delivery platforms are lipid nanoparticles and ligand conjugation approaches. With the progress of exosome biology, exosomes are expected to contribute to the solution of various problems associated with nucleic acid drugs.

## 1. Introduction

Cancer is one of the most life-threatening diseases. Several therapeutic options are available for the treatment of cancer. In addition to the progress made in conventional treatments such as chemotherapy, radiation therapy, and surgery, immunotherapy has recently been developed [1]. However, the ultimate goal of curing cancer has not been achieved for many cancer types, and several medical needs remain unmet.

Currently, most clinically approved drugs including those for cancer are small molecules or protein/antibody-based biologics. Nucleic acid drugs are next-generation drugs, and enormous efforts have been made in developing them. It was 40 years ago since nucleic acid was shown to inhibit virus replication [2]. Thereafter, nucleic acid drugs have been marketed mainly for rare diseases at present by overcoming various challenges and developing strategies with appropriate disease selection.

Herein, the types of nucleic acid drugs and their advantages are reviewed, and the clinical situation is summarized. The challenges in the use of nucleic acid drugs and the relevance of exosomes in overcoming these challenges are also discussed, together with a description of the future prospects.

## 2. Advantages of Nucleic Acid Drugs

Nucleic acid drugs control the biological functions of cells, based on nucleotide sequence information. The functioning of these drugs is either based on their expression in cells or is mediated through the regulation of genes, specifically those having complementary sequences. These mechanisms provide a major advantage in that nucleic acid drugs can be designed regardless of the localization or structure of the target molecule, enabling approaches to target molecules that have not been possible with small molecules or antibodies. Recently, non-coding RNAs such as microRNAs (miRNAs), which are not translated into proteins, have been shown to be involved in several biological processes [3,4,5]. Nucleic acid drugs can also be used to target such molecules.

Another feature of nucleic acid drugs is that once a platform is established, it is possible to create a drug simply by changing the nucleotide sequence of the target gene; thus, rapid and efficient drug development can be expected. After sequencing of the human genome, the relationships between gene mutations and disease phenotypes are being unraveled with the progress in sequencing technology [6,7], and nucleic acid drugs are considered to meet this trend.

For example, in an N-of-1 treatment of a patient diagnosed with Batten disease, the causative gene and its mutations were identified. It took only 10 months from the identification of causal mutation to the development of a nucleic acid drug candidate and its administration to the patient [8]. In the recent COVID-19 pandemic, the mRNA vaccine was first administered to humans within approximately two months of the release of the SARS-CoV-2 genome sequence [9]. Indeed, there are special circumstances such as the Batten disease or the COVID-19 pandemic requiring an early therapeutic intervention; however, the fact that these drugs were developed in such a short period can largely be attributed to them being nucleic acid drugs.

The mode of action of nucleic acid drugs is characteristically different from that of conventional drugs. As will be discussed in detail in the next section, it is possible to inhibit the production of target molecules, which is expected to have a different effect than inhibiting target molecules that have already been synthesized. In addition, a variety of nucleic acid drugs such as new functional proteins have been proposed in which the splicing patterns are changed or mutant proteins are converted to normal proteins through the editing of base sequences.

## 3. Classes of Nucleic Acid Drugs

Nucleic acid drugs have been classified into five categories according to the manner in which they act on target genes (Table 1). Although aptamers [10] and CpG oligonucleotides [11] are considered as nucleic acid drugs, they were not included in this review because they act by recognizing the structure and the sequence information itself is not used. Moreover, gene therapy using vectors such as viruses and plasmids is usually distinct from nucleic acid drugs, whereas mRNA medicine, which has made remarkable progress in recent years, is different from vector-based gene therapy and is included in this review.

### 3.1. Inhibition Type

#### 3.1.1. Antisense Oligonucleotides

An antisense oligonucleotide (ASO) is a short single-stranded 13–30 mer DNA. Using an oligonucleotide with a sequence complementary to that of the target gene, a DNA/RNA double-stranded structure is formed at the targeted site, leading to the inhibition of gene function. Besides the genes for mRNAs, miRNAs can also be targeted, in which case, the ASO targeting the miRNA is referred to as antimir or antagomir [12,13].

ASO not only results in a steric block through the formation of a strong double-stranded structure, but also induces cleavage by RNase H. RNase H is an endogenous enzyme that recognizes DNA/RNA duplexes and specifically cleaves only the RNA strand. Therefore, the formation of duplexes by ASOs can catalyze targeted cleavage by RNase H [14].

Because DNA is unstable, various chemical modifications have been used to ensure the applicability of ASO as a nucleic acid drug. Representative examples of such modifications include those in which the phosphate diester bond is converted to a phosphorothioate bond [15] and those in which a substituent is introduced at the 2′-position of sugar in the nucleic acid [16]. 2′-O-methyl, 2′-O-methoxyethyl, and 2’-fluoro are the most common modifications of nucleic acids with sugar moieties. Moreover, 2′-O,4′-C-methylene-bridged nucleic acid/locked nucleic acid (2’,4’-BNA/LNA) [17], 2′-O,4′-C-ethylene-bridged nucleic acid (ENA) [18], and 2’,4’-constrained 2’-O-ethyl (constrained ethyl) BNA (cEt) [19] enhance both nuclease stability and binding affinity toward target RNA. Because phosphorothioate modification is highly resistant to nucleases and is recognized by RNase H, it is often introduced at all positions in the ASO. In contrast, sugar-modified nucleic acids are expected to contribute to the formation of a strong double-strand due to their high RNA binding strength; however, they are not recognized by RNase H [20]. Therefore, the gapmer structure is often used, in which sugar-modified nucleic acids are placed at both ends of the ASO, and natural DNA is placed in the center.

In addition to chemical modifications, a DNA/RNA heteroduplex oligonucleotide format has also been proposed [21,22]. This consists of an antisense DNA strand and a complementary sense RNA strand, which not only improves nuclease resistance but also allows various delivery molecules to be conjugated to the sense RNA strand. The sense RNA strand is cleaved by RNase H upon uptake by the cell and acts in the same way as the antisense strand.

ASOs were studied earlier than other nucleic acid drugs. Fomivirsen, an ASO, was approved as the first nucleic acid drug in the United States in 1998 [23]. Although it is not a gapmer-type drug, the subsequently approved mipomersen [24] and inotersen [25] are gapmer-types.

#### 3.1.2. siRNAs

siRNAs are double-stranded RNAs with each strand being 20–30 nucleotide long. Research on siRNAs originated with the discovery of RNA interference [26]. When an siRNA is taken up by a cell, it forms a complex called the RNA-induced silencing complex (RISC) in the cytoplasm [27]. The silencing activity of siRNA is determined by factors such as the ease with which RISC is formed and the strand of the double-stranded RNA that is ultimately used as the complementary strand to the mRNA. Several conditions determining the effectiveness of siRNA sequences have been proposed based on the results of in vitro experiments [28,29].

As with ASOs, modified nucleic acids are also used in siRNAs to suppress nuclease degradation and to avoid inducing immune responses; however, RNAi activity may be lost if modifications are inappropriately placed within the siRNA sequence [30]. It is important to determine how to position the modified nucleic acid in order to maintain the RNAi activity. For example, Allerson et al. showed that a fully modified siRNA consisting of alternating 2‘-OMe and 2‘-F nucleotides had enhanced potency and stability [31].

The clinical application of siRNA drugs is rapidly expanding. Patisiran was the first siRNA drug approved by the U.S. Food and Drug Administration (FDA) in 2018 [32], and three more drugs were approved in 2020.

### 3.2. Splice Switching Type

Splice switching oligonucleotide (SSO) is a form of single-stranded DNA, the same as ASOs, which controls the splicing of pre-mRNA, the precursor of mature mRNA. By designing a sequence that is complementary to the exon–intron junction sequence of pre-mRNAs, it is possible to form a double-stranded DNA and artificially control the splicing of specific exons [33]. There are two main methods to control splicing: skipping, which excludes exons that would normally be incorporated, and inclusion, which prevents exons that would normally be removed from being excluded. Eteplirsen [34], which was approved in 2016, is a skipping type drug, and nusinersen [35,36], which was also approved in 2016, is an inclusion type.

Similar to ASOs, modified nucleic acids are also used for SSOs. In the case of SSO, modified nucleic acids are often used for the central part of the oligonucleotide because the RNA/DNA duplex does not need to be cleaved by RNase H, and it is not necessary to have the gapmer structure. In some cases, morpholino nucleic acid [37], which is an uncharged nucleic acid, is used as a modified nucleic acid.

### 3.3. Editing Type

RNA editing is an intrinsic mechanism in which a DNA sequence is converted at the RNA level [38,39]. Using this mechanism, it is possible to rewrite the sequence information. The most common type of RNA editing in humans is A-to-I RNA editing, in which adenosine (A) is converted to inosine (I) by hydrolytic deamination. Because the inosine residue on an mRNA is recognized as guanosine during translation, this editing process results in the replacement of adenosine by guanosine. The key factor in A-to-I RNA editing is an enzyme called adenosine deaminase acting on RNA (ADAR). Editing oligonucleotides (EON) are artificially designed guide RNAs that are recognized by endogenous ADARs to induce A-to-I RNA editing. It is important to incorporate a function to recruit ADARs and to recognize the sequence to be replaced by the guide RNA, and several methodologies have been reported for this [40,41,42].

An alternative approach that combines the catalytic domain of ADAR protein with guide RNA and introduces both into cells has also been proposed [43,44]; however, this approach is expected to face increasing hurdles in terms of protein preparation and intracellular delivery.

There are no approved RNA-edited nucleic acid drugs as yet, but sepofarsen, indicated for Leber congenital amaurosis and developed by ProQR Therapeutics, is in the most advanced clinical stage [45].

### 3.4. Augmentation Type

In contrast to ASO and siRNA molecules, small activating RNA (saRNA) molecules that enhance the expression of target genes have also been reported [46,47]. These are double-stranded RNAs that promote transcription from a target gene through a sequence designed to recognize an upstream region of the target gene and recruit transcription factors to the target gene on genomic DNA. Because saRNA molecules are similar to siRNAs, it is expected that knowledge obtained from the modification of siRNAs can be used for saRNAs. There is no approved drug of this type as yet, but the first saRNA drug targeting C/EBP-a is in a phase 1 clinical trial for the treatment of hepatocellular carcinoma [48,49].

### 3.5. Replacement Type

#### 3.5.1. miRNA Mimics

An miRNA mimic is a synthetic, double-stranded RNA designed to restore a body function impaired by a decrease in the expression of an miRNA [50]. This double-stranded RNA consists of a guide strand that is completely identical to the endogenous mature miRNA sequence and a passenger strand that is partially or fully complementary to the guide strand. As with siRNAs, miRNA mimics are usually chemically modified. The position and type of modified nucleic acids are considered so that the function of the miRNA is not impaired, because an miRNA forms the RISC complex to exert its function.

Clinically, the miR-34 mimic developed by Mirna Therapeutics is in the most advanced stage of development. miR-34 is known to be a tumor suppressor, because it is involved in the p53 DNA repair pathway [51,52]. The miR-34 mimic was tested in patients with advanced solid tumors; however, it was discontinued due to severe immune-mediated adverse events [53,54]. In addition to miR-34, replacement therapies for miR-29 and miR-16 have also been implemented in clinical applications [55,56].

#### 3.5.2. mRNA Drugs

The mRNA drugs are directly introduced into the target cells and are expressed as proteins without using a vector system [57,58,59]. These drugs are composed of hundreds to thousands of nucleotides, and are different from the category of oligonucleotide-based drugs. They do not need to be delivered to the nucleus, obviating concerns about their insertion into the genome; these drugs are, therefore, expected to be highly safe. However, the effect of these drugs is transient whereas gene therapy approaches work for several years. Taking advantage of these characteristics, clinical applications of mRNA drugs in the field of infectious diseases and cancer vaccines [60] are being explored. In particular, since the onset of the COVID-19 pandemic in 2020, clinical trials of vaccines against SARS-CoV-2 have progressed rapidly, and two mRNA vaccines have been approved for emergency use [61,62].

Like other nucleic acid drugs, mRNA drugs also employ modified nucleic acids so that they are not easily degraded when injected into the body. Furthermore, because an mRNA introduced from outside the cell has strong immunogenicity, modified nucleic acids are also used to avoid such reactions. For example, it is known that replacing uridine in mRNA with pseudouridine suppresses immunogenicity and mRNA degradation, leading to highly efficient translation of the target protein [63].

In addition, mRNA needs to be recognized by molecules involved in the initiation of translation when introduced into the cell. The 5′-Cap structure is usually attached to the 5′-end of natural mRNAs and is essential for the translation process [64]. The mRNA is synthesized in a cell-free system by in vitro transcription from a DNA template, and the direction of addition of the Cap was not controlled well previously. The development of the ARCA method has made it possible to enhance the synthesis and purification of RNA with a 5′-Cap structure [65]. Other innovations have been made in various areas such as codon optimization in the translation region and control of polyA sequence length [66,67].

## 4. Current Status of Clinical Application of Nucleic Acid Drugs

The nucleic acid drugs approved by the FDA and/or the European Medicines Agency (EMA) are listed in Table 2. As of June 2021, 15 drugs were approved. The first nucleic acid drug was an ASO type approved in 1998, which was followed by the SSO type in 2016 and siRNA drugs in 2018, and mRNA drugs in 2020. Table 3 shows nucleic acid drugs that are currently in phase 3 clinical trials.

Many of the target diseases are rare diseases for which the relationship between the genetic mutation and the disease is clear. No nucleic acid drugs have been approved for the treatment of cancer.

### 4.1. Target Tissues of Nucleic Acid Drugs

For clinical application of nucleic acid drugs, the most important issue is the delivery of nucleic acids to target tissues and cells. Nucleic acids are not easily taken up by cells due to their highly hydrophilic, polyvalent anionic properties. When administered intravenously, they tend to accumulate in the liver and kidneys [68,69]. Therefore, nucleic acid drugs without any drug delivery system (DDS), which have been approved or are in the late stage of clinical trials, are focused either on local administration or on targeting of the liver and kidneys after systemic injection.

Examples of tissues targeted for local administration are the eye and central nervous system. Fomivirsen is intended to act on the retina through intravitreal administration [23], and nusinersen is a representative drug that can be delivered to the central nervous system through intrathecal administration [70].

Liver is the most targeted tissue for systemic administration. Mipomersen, administered subcutaneously, causes gene suppression in the liver [24]. Teprasiran, commonly known as QPI-1002, is intended to act on the kidney after intravenous administration [71,72,73].

Many attempts have been made to combine nucleic acids with a DDS to protect them from degradation and to ensure their delivery to the target cells. The details of such attempts are described by Yamayoshi et al. in this Special Issue [74]. The DDS used in the approved nucleic acid drugs include lipid nanoparticles (LNP) and GalNAc conjugation.

LNPs are lipid-based nanoparticles that encapsulate nucleic acids [75]. When injected intravenously, LNPs diffuse rapidly and are passively targeted to the liver [76], making them suitable for delivering nucleic acids to the liver. They have also been reported to bind to apolipoprotein E (apoE) in the blood and are taken up by hepatocytes expressing apoE receptors [77]. The siRNA drug, patisran, targets the TTR gene, which is expressed in the liver. Patisiran is formulated in LNP that contains cholesterol, DSPC (a polar lipid), DLin-MC3-DMA (an ionizable lipid), and PEG-lipid [78]. LNPs can be further modified with peptides, PEG, and other ligands to extend their retention time in the blood or to target specific cells [79,80,81].

Two approved SARS-CoV-2 mRNA vaccines also use LNPs as DDS. Previous preclinical and clinical data have shown that these LNP-formulated mRNA vaccines, administered through intramuscular injection, are taken up by myocytes and dendritic cells, where the mRNA is translated, followed by subsequent activation of the immune response [82,83]. An attempt to clinically deliver naked mRNA without using any DDS has been made for cancer vaccines [60,84]. The major mechanism of cellular uptake of naked mRNA is micropinocytosis [85,86].

GalNAc is a ligand for the asialoglycoprotein receptor, which is highly expressed on hepatocytes [87]. This ligand has been directly conjugated with nucleic acids and shows effective delivery to and internalization into hepatocytes [88,89]. GalNAc conjugate technology has been adapted for both siRNAs and ASOs. siRNA conjugates such as givosiran [90] and lumasiran [91] have been approved whereas ASO conjugates have been clinically tested. When using chemical conjugation, the nucleic acid portion is directly exposed to serum, and therefore, protection from degradation is more important than when using LNPs. In GalNAc-siRNAs such as givosiran and lumasiran, all nucleotides are modified such as 2′-F-RNA and 2′-OMe-RNA instead of using the unmodified RNA.

### 4.2. Competition for the Same Target

In recent years, many nucleic acid drugs have been launched in the market, and there has been competition for the same targets. In particular, the targeting of the liver by nucleic acid drugs is a conspicuously competitive area, with multiple formats being investigated in clinical stages; these include ASO alone, GalNAc-ASO, LNP-siRNA, and GalNAc-siRNA formats. Inotersen (in the ASO format) and patisiran (in the LNP-siRNA format) were approved in 2018 [25,32] to target the TTR gene expressed in the liver. The indication for these drugs is hereditary transthyretin-mediated amyloidosis, in which the TTR protein destabilized by mutations is deposited extracellularly as fibrillar insoluble amyloids, causing organ dysfunction [92]. Both drugs exert their therapeutic effects by inhibiting the production of TTR. The results of phase 3 clinical trial showed that patisiran had a stronger effect on mNIS+7, an index of drug efficacy, with an improvement of 34.0 points for patisiran compared to only 19.7 points for inotersen [93,94]. Patisiran must be administered intravenously, whereas inotersen can be injected subcutaneously. Therefore, patisiran and inotersen can be used according to the clinical situation. Currently, Alnylam is developing liver-targeted siRNA drugs in a GalNAc-conjugate format other than patisiran, and vutrisiran, a GalNAc-conjugation siRNA drug for TTR, is in the phase 3 stage. It is assumed that GalNAc-siRNA can be administered subcutaneously, unlike LNP-siRNA, and therefore, the development of GalNAc conjugates is expected to increase.

Besides the liver targeting nucleic acids, there is competition in Duchenne muscular dystrophy (DMD) therapeutics among SSO-type nucleic acid drugs. Although many such drugs are used in different ways depending on the position of mutation in a patient, there is competition in some cases. For example, two approved drugs, golodirsen [95] and viltolarsen [96], are amenable for dystrophin exon 53 skipping. In a clinical trial, viltolarsen increased the dystrophin levels to 5.9% of the normal levels after 24 weeks of treatment in eight patients receiving 80 mg/kg dose, an increase of 5.3 percentage points from the baseline [97]. Administration of golodirsen resulted in less than one percentage point improvement over the 48-week treatment period [98]. However, the number of patients treated with both drugs was small, and more data are expected to be added in the future.

There is competition not only for the same target among nucleic acid drugs, but also with regard to other modalities. Inclisiran, a GalNAc-siRNA drug targeting PCSK9, has been approved by the EMA [99], but PCSK9-targeting antibody drugs, evolocumab and alirocumab, were already approved [100]. These antibody drugs are administered once every 2–4 weeks. In a clinical trial of inclisiran, a single subcutaneous dose of 300 mg lowered LDL cholesterol levels by 38% after 180 days, which is different from the durable inhibitory effect obtained with antibody drugs [101]. In fact, the clinically recommended dose of inclisiran is 284 mg, administered subcutaneously as a single injection on day 1, day 90, and every six months, thereafter [102].

A similar case has been reported for drugs targeting complement C5. C5 monoclonal antibody drugs such as eculizumab and ravulizumab have been approved for PNH and aHUS [103,104], whereas cemdisiran, a GalNAc-siRNA targeting the C5 gene, is in a phase 2 clinical trial [105,106]. This trend of modality conversion from antibody drugs is expected to continue in the future.

Furthermore, there have been cases where approved nucleic acid drugs have been applied to other modalities. Nusinersen is an SSO-type nucleic acid drug approved for the treatment of spinal muscular atrophy (SMA) [36]. It is designed to replace the mutated SMN1 function by regulating the splicing of *SMN2*, a homolog of *SMN1*, to express a protein with a function similar to that of SMN1. In contrast, a gene therapy drug, onasemnogene abeparvovec (brand name, Zolgensma), which is administered via the AAV vectors encoding the normal SMN1 gene, was approved by the FDA in 2019 [107,108]. Onasemnogene abeparvovec can be administered intravenously because the AAV9 vector enables delivery across the blood–brain barrier [109,110]. In general, AAV vector treatment is administered only once in a lifetime because this approach induces neutralizing antibodies against AAV; however, non-clinical data show persistent expression in neurons [111]. Nusinersen requires intrathecal injections of four loading doses followed by maintenance doses every four months thereafter [35]. Considering this situation, gene therapy appears advantageous. However, it remains unknown whether persistent expression can be maintained in humans, and further data on long-term safety and efficacy are required. In addition, the combination therapy of nusinersen and onasemnogene abeparvovec is being investigated clinically [112].

The approach of drug development with different modalities for the same target is likely to continue. Nucleic acid drugs cannot be expected to work for several years, unlike gene therapy; however, they can be administered at the required time and in the required dose. Taking advantage of these characteristics, in the future, nucleic acid drugs will be used for the treatment of diseases where the number of doses and the dosage can be changed flexibly according to the patient’s situation.

## 5. Challenges in Nucleic Acid Drugs

Nucleic acid drugs have outstanding properties not found in other modalities, but also have many challenges. In addition to the low stability of the nucleic acid itself, these include rapid clearance from the blood circulation, delivering to target cells, inefficient uptake of the nucleic acid into cells, and immunogenicity of the nucleic acid. As mentioned in the previous section, most of the nucleic acid drugs that have been approved or are undergoing phase 3 trials are either locally administered (including intrathecal administration) or hepatic delivered via systemic circulation, and various studies are still underway to overcome the challenges associated with the delivery of nucleic acid drugs to other tissues such as cancer. In this section, I focus on the systemic circulation type of nucleic acid drugs.

### 5.1. Pharmacokinetics, Delivery Efficiency, and Safety of Nucleic Acid Drugs

Nucleic acids themselves are easily degraded by enzymes, but this is being overcome by the development of modified nucleic acids, as described in Section 3. It is also known that phosphorothioate modified nucleic acids, which are relatively commonly used in nucleic acid modification, have the effect of increasing protein binding [113]. Normally, natural nucleic acids, due to their molecular weight and size, are filtered out by the glomeruli of the kidney and excreted in urine, however, when bound to serum proteins, they escape glomerular filtration. Therefore, phosphorothioate modified nucleic acids increase their retention in the blood and prolong their elimination half-life. In fact, the elimination half-life of phosphorothioate modified ASO in clinical applications has been reported to be several weeks [114].

When nucleic acids are administered intravenously or subcutaneously, they are mainly distributed to the liver and kidneys, but are also widely distributed to other tissues. However, they are not delivered to the brain, and it is thought to be difficult for them to cross the blood–brain barrier. Given these characteristics, in order to act on nucleic acids besides the liver and kidneys, it will be necessary to improve a delivery method to be more selective to specific cells.

GalNAc-conjugated nucleic acids are more efficiently taken up by hepatocytes, allowing effective concentrations in hepatocytes to be achieved at lower doses compared to unconjugated nucleic acids, with much lower exposure in plasma and extra-hepatic tissues (over 50–100-fold) [115]. This results in higher plasma clearance and a wider safety margin without having to worry about high C max, which is often considered as a cause of non-target related toxicities for unconjugated nucleic acids.

In order to deliver nucleic acids to extra-hepatic cells, the use of ligands such as GalNAc can be considered. GalNAc is recognized by the asialoglycoprotein receptor (ASGPR), which is highly expressed in hepatocytes at levels as high as 500,000 copies/cell and cycles as quickly as 10–15 min [116]. Unfortunately, no other receptor–ligand combination has been found that exhibits these properties. However, it is said that less than 0.01% of GalNAc-conjugate nucleic acid escapes from endosomes after being taken into cells, and the nucleic acid is not effectively utilized [116]. By approaching this point, there is a possibility of overcoming the problem of cell delivery.

For example, endosome-disrupting agents can be bound to nucleic acids or encapsulated in nanoparticles. Chloroquine and peptides with such properties have been reported [117,118], but they also work against other intracellular endosomes and may be cytotoxic. Therefore, there is a need for molecules that are less toxic and can escape from endosomes more efficiently.

Nucleic acid delivery by nanoparticles has also contributed to overcoming various challenges associated with nucleic acid drugs. There are several types of nanoparticles including lipid-based, polymer-based, inorganic-based, and nucleic acid-based, all of which aim to deliver nucleic acids to target cells by protecting them from degradation and avoiding renal excretion. The mechanism of endosomal escape by nanoparticles is described in detail in a review by Smith et al. [119] and includes membrane fusion with the cell membrane, osmotic effect, swelling of nanoparticles, and membrane destabilization.

It should also be noted that nucleic acids have an off-target effect by acting on innate immune cells. Pattern recognition receptors (PRRs) in endosomes or cytosol of the cells are activated by nucleic acids and show responses such as cytokine secretion [120]. Some specific sequence motifs and nucleic acid modifications as well as combinations thereof, have been reported to have immunostimulatory properties. Therefore, it is effective to reduce immune stimulation by designing sequences, modifications, and structures that are not recognized by PRRs. However, the types and localization of PRRs have not been elucidated to explain all immune stimuli, so a systematic approach including various measures is desirable rather than undertaken solely.

For instance, phosphorothioate modified nucleic acid is one of the modified nucleic acids that have immunostimulatory properties in combination with specific sequences. In clinical trials, adverse effects after administration of phosphorothioate ASO include fever and fever-like reactions, prolonged activated partial thromboplastin time, and thrombocytopenia [121,122,123]. Sequence optimization and the introduction of different modified nucleic acid has led to decreased immune stimulation.

### 5.2. Challenges in the Field of Cancer Therapeutics

No nucleic acid drugs have been approved for the treatment of cancer; some ASOs have advanced to phase 3 trials but have not been approved, and siRNAs have not yet advanced beyond phase 2.

ASOs that have advanced to phase 3 include oblimersen targeting bcl2 [124], custirsen targeting clusterin [125], and trabedersen targeting FGFβ2 [126], all of which were administered by intravenous administration without any DDS. None of them have reached the primary endpoint in phase 3 trials, and development has been discontinued or is being rechallenged in phase 1 trials. For example, there was no significant difference in overall survival between curtirsen combined with abazitaxel/docetaxel and prednisone versus chemotherapy alone in metastatic castration-resistant prostate cancer [125]. However, the ASOs that have completed phase 3 are first-generation ASO drugs, and future trials using second-generation ASOs may lead to increased efficacy and improved overall survival and progression-free survival.

In the case of siRNAs, they are all formulated in combination with some DDS. So far, CALAA-01 [127], TKM-PLK1 [128], Atu027 [129], ALN-VSP02 [130], siG12D-LODER [131], and so on have been clinically administered. Most are LNPs, lipid-based nanoparticle, but CALAA-01 is a cyclodextrin-based nanoparticle coated with human transferrin, and siG12D-LODER is based on a biodegradable polymer.

All were found to be acceptably safe in the dose range of the phase 1 study, and the maximum tolerated dose was not determined except for TKM-PLK1 at 0.75 mg/kg. Many adverse events are also limited to cytokine elevation and so on. Serious hepatotoxicity and nephrotoxicity have rarely been reported, although one patient with pancreatic neuroendocrine tumor and a history of splenectomy and hepatectomy developed hepatic failure and subsequently died after receiving 0.7 mg/kg of ALN-VSP02. In contrast, ALN-VSP02 has shown promising results, with one patient achieving a complete response and the others having stable disease at all sites for approximately 8–12 months.

The results of the clinical trials are as described above, but most of the products have not moved forward and only siG12D-LODER is currently in phase 2. Many companies have explained that their reason for this is that they are reviewing their business portfolio. This may also be due to the fact that it is difficult to predict the final clinical efficacy based on the results of phase 2 trials.

Not only limited to nucleic acid drugs, in general, the success rate of a product that has entered clinical trials for cancer treatment is lower than that of products for other diseases [132]. One of the reasons is the difference in the confirmation of drug efficacy between non-clinical and clinical trials, specifically, the degree to which non-clinical models reflect the tumor environment in clinical trials, and the correlation between the rate of tumor volume change in non-clinical models and clinical survival endpoints. Nucleic acid drugs, in particular, must be delivered to cancer cells, and their efficacy has been confirmed in non-clinical studies. In clinical practice, for example, siRNA has been detected in the tumor tissues of patients treated with ALN-VSP02, and the decrease in expression of target genes has been confirmed [131]. However, due to the heterogeneity of cancer cells in tumors, it is difficult to rigorously verify whether the decreased expression of target genes is the effect of siRNA delivery to the cancer cells, making it difficult to determine the technical improvement of the drug and its level based on the clinical results.

Many clinically used products, whether ASOs or siRNAs, have been modified to increase their retention in the bloodstream for passive targeting to cancer cells, to escape immune cell uptake, and to act through tumor blood vessels, but it is difficult to determine to what extent these improvements have contributed to clinical success. In contrast, an alternative approach is active targeting with a combination of ligands. In this strategy, the target cells are clear, and the clinical effects is relatively predictable based on the expression information in tumor tissues. In addition to formats in which antibodies or small molecules such as folic acid are directly bound to nucleic acids, formats in which nanoparticles with targeting elements are arranged are also being investigated. One such example is CALAA-01, which is functionalized with a human transferrin ligand that targets the transferrin receptor, which is highly expressed in tumor cells [127]. In patients treated with CALAA-01, the presence of nanoparticles only in tumor cells has been confirmed by staining [133]. However, the existence of transferrin has also raised new challenges including formulation instability and the occurrence of major dose-limiting toxicities in two of the five patients enrolled in the trial.

As another approach, a smart nanocarrier that responds to the external environment and stimuli is being investigated. These nanocarriers have the ability to respond to pH and metabolites in the tumor environment as well as to light and magnetic field stimuli [134]. Although it is still in the preclinical stage, it is expected to reduce the uncertainty surrounding the drug development for cancer, especially when combined with external interventions such as radiotherapy.

## 6. Overcoming Challenges Associated with Nucleic Acid Drugs by Using Exosomes

### 6.1. Delivery of Nucleic Acids

As mentioned in the previous section, the biggest challenge with nucleic acid drugs is their delivery to the target tissue. With the progress in nucleic acid modification and in the development of excellent DDS such as LNPs and GalNAc-conjugates, many nucleic acid drugs have been used clinically. However, delivery of nucleic acids is still not easy for many tissues and cells including cancer cells.

Exosomes are a class of extracellular vesicles consisting of lipid bilayers with a diameter of approximately 100 nm and are thought to be responsible for intercellular communication [135,136]. Recently, exosomes have been expected to be used as a DDS and to solve the hurdle of delivering nucleic acid drugs. In particular, molecular analyses have indicated that the metastatic destination of a cancer is determined by exosomes derived from cancer cells, and its organ specificity is determined by integrins contained in the exosomes [137]. By utilizing the intrinsic properties, it is expected that the same effect will be achieved in the clinical use, and this property can be used for the delivery of drugs to target cancer cells. Since exosomes can pass through the BBB and have low immunogenicity, so they can be used in a variety of ways other than delivery to cancer cells. There are also established methodologies such as the presentation of molecules on the surface of exosomes. Clinical trials of exosome drugs started last year [138].

Incubation [139], sonication [140], electroporation [141], and liposome–exosome hybrids [142] have been reported as methods for incorporating target nucleic acids into purified exosomes. Detailed explanations regarding these methods have been provided by Yamayoshi et al. in this special review [74].

There are various types of exosomes, and it is difficult to assume that all the substances contained in the exosomes are uniform, even if they are derived from the same cell. Regulations such as standardization are expected to be discussed in the future, but at present, methods for purifying exosomes from milk and from cultured cells are being considered.

Another issue that awaits further progress is the long-term storage of exosomes. Although cryopreservation is currently common, it is important to prevent ice crystals from forming [143].

### 6.2. Approaches for Multifactorial Diseases

To date, all approved nucleic acid drugs target only a single gene. This is based on the idea that a disease is caused by a single genetic abnormality and that the disease can be treated by controlling the expression of that gene. However, there are a variety of diseases in which multiple gene mutations are involved, and the discovery of drugs that target multiple genes instead of a single target has been proposed [144]. In particular, cancer drugs and combination therapies that target multiple signals are being explored [145].

Although it is a fact that nucleic acids can only act on a single gene given their nature, an approach in which LNPs encapsulate nucleic acids against multiple targets is one of the possible solutions. In fact, ALN-VSP, which contains two siRNAs against VEGF and KSP in a single LNP, was clinically tested for liver cancer therapy [130]. The same concept applies not only to siRNAs, but also to ASOs and mRNAs. Moreover, exosomes can be used instead of LNPs, as mentioned earlier.

### 6.3. Pharmacodynamic (PD) Marker of Nucleic Acid Therapeutics

To confirm whether a nucleic acid drug has worked on the target tissue, it is a common practice to remove the target tissue and check the changes in the expression of the target genes. However, in many cases, it is not easy to remove the tissues, and it is difficult to measure the changes over time. Although the use of other indicators of efficacy is a realistic option, it is desirable to have a method to directly measure the expression of target genes without removing the tissues.

Sehgal et al. showed that liver-derived RNA circulates in the bloodstream, and when siRNA against genes expressed in the liver is administered, there is a correlation between the decrease in the expression of the target gene in the liver and the decrease in the expression of the target gene as determined in blood-derived RNA [146]. Based on this correlation, givosiran, a GalNAc-siRNA targeting ALAS1, was monitored by extracting RNA from exosomes in the blood and urine [147,148]. This method is attracting attention as a new PD marker.

Although this approach has not yet been put to practical use for genes expressed, except in the liver, it will be a revolutionary method if gene expression in tissues can be monitored by isolated exosomes from blood or urine. It is expected to increase the information obtained from clinical trials and contribute to the improvement in technology related to nucleic acid medicine, and future research is required as a new application of exosomes.

**Figure 1:**
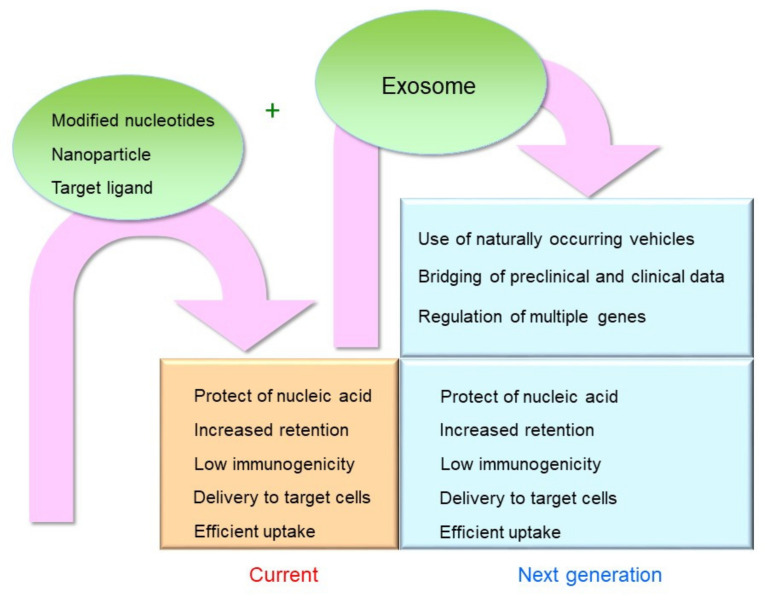
Current status of overcoming challenges and future expectations for exosomes.

Finally, Figure 1 summarizes the issues that are currently required for nucleic acid drugs and those that are expected to be solved by exosomes in the future.

## 7. Conclusions and Perspectives

Nucleic acid drugs operate via a wide variety of mechanisms. With the progress in the development of DDS and chemical modification of nucleic acids, many drugs have already been approved as pharmaceuticals. By selecting targets that take advantage of the characteristics of nucleic acid drugs, which are different from those of conventional ones, nucleic acid drugs are being developed for diseases such as the rare ones for which no therapeutic agents exist. Nucleic acid drugs are being established as the third modality besides small molecule and antibody drugs.

To date, no nucleic acid drug has been approved for the treatment of cancer. It is hoped that further understanding of the biology of exosomes will enable us to overcome the challenges associated with nucleic acid drugs, and new therapeutic options will increase in the near future.

## Figures and Tables

**Table 1:** Classification of nucleic acid drugs.

Regulation Type	Name	Structure	Length (nt)	Representative Companies
Inhibition	ASO	ssDNA	13–30	Ionis, Nippon Shinyaku
	siRNA	dsRNA	20–30	Alnylam, Dicerna, Quark
Splice switching	SSO	ssDNA	20–30	Sarepta, Ionis
Editing	EON	ssRNA	20–40	ProQR
Augmentation	saRNA	dsRNA	20–30	MiNA
Replacement	miRNA mimic	dsRNA	20–30	Mirna, miReven
	mRNA	ssRNA	hundreds to thousands	BioNTech, Moderna

ssDNA, single strand DNA; dsRNA, double strand RNA, ssRNA, single strand RNA.

**Table 2:** Nucleic acid drugs approved by the FDA or EMA (as of 30 June 2021).

Drug Name	Year of Approval	Type	Target	Indication	DDS	Company
Fomivirsen	1998	ASO	CMV IE2	Cytomegalovirus retinitis	Naked	Ionis
Mipomersen	2013	ASO	ApoB-100	Homozygous familial hypercholesterolemia	Naked	Ionis
Eteplirsen	2016	SSO	Exon 51 of DMD	Duchenne muscular dystrophy	Naked	Sarepta
Nusinersen	2016	SSO	Exon 7 of SMN2	Spinal muscular atrophy	Naked	Ionis
Inotersen	2018	ASO	TTR	Hereditary transthyretin mediated amyloidosis	Naked	Ionis
Patisiran	2018	siRNA	TTR	Hereditary transthyretin mediated amyloidosis	LNP	Alnylam
Golodirsen	2019	SSO	Exon 53 of DMD	Duchenne muscular dystrophy	Naked	Sarepta
Volanesorsen	2019	ASO	ApoC3	Familial chylomicronemia syndrome	Naked	Ionis
Givosiran	2019	siRNA	ALAS1	Acute hepatic porphyria	GalNAc	Alnylam
Viltolarsen	2020	SSO	Exon 53 of DMD	Duchenne muscular dystrophy	Naked	Nippon Shinyaku
Lumasiran	2020	siRNA	hydroxyacid oxidase 1	Primary hyperoxaluria type 1	GalNAc	Alnylam
Inclisiran	2020	siRNA	PCSK9	Familial hypercholesterolemia	GalNAc	Alnylam, Novartis
Tozinameran	2020	mRNA	SARS-CoV-2	COVID-19 Vaccine	LNP	BioNTech, Pfizer
Elasomeran	2020	mRNA	SARS-CoV-2	COVID-19 Vaccine	LNP	Moderna
Casimersen	2021	SSO	Exon 45 of DMD	Duchenne muscular dystrophy	Naked	Sarepta

**Table 3:** Nucleic acid drugs in a phase 3 trial (as of 30 June 2021).

Drug Name	Type	Target	Indication	DDS	Company
Pelacarsen	ASO	Apolipoprotein A	Cardiovascular disease	GalNAc	Ionis, Novartis
Eplontersen	ASO	TTR	TTR amyloidosis	GalNAc	Ionis
APOCIII-LRx	ASO	ApoC3	Familial chylomicronemia syndrome	GalNAc	Ionis
Tofersen	ASO	SOD1	Amyotrophic lateral sclerosis	Naked	Ionis, Biogen
Tominersen	ASO	HTT	Huntington’s disease	Naked	Ionis, Roche
ION-363	ASO	FUS	Amyotrophic lateral sclerosis	Naked	Ionis
Nexagon	ASO	Connexin 43	Corneal injury	Naked	Ocunexus
Fitusiran	siRNA	Antithrombin III	Hemophilia	GalNAc	Alnylam, Sanofi
Vutrisiran	siRNA	TTR	TTR amyloidosis	GalNAc	Alnylam
Nedosiran	siRNA	LDHA	Primary hyperoxaluria	GalNAc	Dicerna
Teprasiran	siRNA	P53	Acute kidney injury	Naked	Quark
Cosdosiran	siRNA	Caspase 2	Non-arteritic anterior ischemic optic neuropathy	Naked	Quark
Tivanisiran	siRNA	TRPV1	Dry eye	Naked	Sylentis
Sepofarsen	EON	CEP290	Leber congenital amaurosis	Naked	ProQR
Zorecimeran	mRNA	SARS-CoV-2	COVID-19 vaccine	LNP	CureVac
